# Application of CRISPR/Cas9 Genome Editing Technology for the Improvement of Crops Cultivated in Tropical Climates: Recent Progress, Prospects, and Challenges

**DOI:** 10.3389/fpls.2018.00617

**Published:** 2018-05-08

**Authors:** Effi Haque, Hiroaki Taniguchi, Md. Mahmudul Hassan, Pankaj Bhowmik, M. Rezaul Karim, Magdalena Śmiech, Kaijun Zhao, Mahfuzur Rahman, Tofazzal Islam

**Affiliations:** ^1^Department of Biotechnology, Bangabandhu Sheikh Mujibur Rahman Agricultural University, Gazipur, Bangladesh; ^2^Institute of Genetics and Animal Breeding of the Polish Academy of Sciences, Jastrzębiec, Poland; ^3^Division of Genetics, Genomics and Development School of Biosciences, The University of Melbourne, Melbourne, VIC, Australia; ^4^Department of Genetics and Plant Breeding, Patuakhali Science and Technology University, Patuakhali, Bangladesh; ^5^National Research Council of Canada, Saskatoon, SK, Canada; ^6^Department of Biotechnology and Genetic Engineering Jahangirnagar University Savar, Dhaka, Bangladesh; ^7^National Key Facility for Crop Gene Resources and Genetic Improvement, Institute of Crop Science, Chinese Academy of Agricultural Sciences, Beijing, China; ^8^Extension Service, West Virginia University, Morgantown, WV, United States

**Keywords:** CRISPR/Cas9, genome editing, tropical crops, climate change, blast, disease, genetic engineering, food security

## Abstract

The world population is expected to increase from 7.3 to 9.7 billion by 2050. Pest outbreak and increased abiotic stresses due to climate change pose a high risk to tropical crop production. Although conventional breeding techniques have significantly increased crop production and yield, new approaches are required to further improve crop production in order to meet the global growing demand for food. The Clustered Regularly Interspaced Short Palindromic Repeats (CRISPR)/Cas9 (CRISPR-associated protein9) genome editing technology has shown great promise for quickly addressing emerging challenges in agriculture. It can be used to precisely modify genome sequence of any organism including plants to achieve the desired trait. Compared to other genome editing tools such as zinc finger nucleases (ZFNs) and transcriptional activator-like effector nucleases (TALENs), CRISPR/Cas9 is faster, cheaper, precise and highly efficient in editing genomes even at the multiplex level. Application of CRISPR/Cas9 technology in editing the plant genome is emerging rapidly. The CRISPR/Cas9 is becoming a user-friendly tool for development of non-transgenic genome edited crop plants to counteract harmful effects from climate change and ensure future food security of increasing population in tropical countries. This review updates current knowledge and potentials of CRISPR/Cas9 for improvement of crops cultivated in tropical climates to gain resiliency against emerging pests and abiotic stresses.

## Introduction

Population growth, climate change, and food shortage are some of the threatening current issues for the world community. World population is growing rapidly and is projected to reach 9.7 billion by 2050 (Clarke and Zhang, 2013). The rate of population growth is higher in the tropical countries than the countries in temperate regions. Seven among the nine countries where more than 50% population growth are expected between now and 2050 are in the tropical region. These countries are India, Nigeria, Democratic Republic of the Congo, Ethiopia, United Republic of Tanzania, Indonesia and Uganda. It has been estimated that Nigeria will be the largest country in terms of population after India and China by 2050 (Campos and Caligari, 2017). More than two third of the people who are in extreme poverty live in tropical countries. The ability to feed this rapidly growing population in the tropical region will soon become a critical issue that will need to be addressed by the society. This situation will be exacerbated due to decrease in available arable land coupled with reduced crop yields that are both predicted to arise from the climate change. For instance, the International Rice Research Institute (IRRI) has estimated that one hectare of cultivable land is lost every 7.7 s, and the rate of loss may accelerate with increased global temperatures (Stamm et al., 2011). Climate change not only affects crop production through altered weather patterns, but also via increased environmental stresses such as soil salinity, drought and emergence of new disease and insect-pests. To keep up with the pace of population growth, it has recently been estimated that food production will need to be increased by 50% by 2030 and by 70–100% by 2050 for a well-fed world population (Godfray et al., 2010; Jones et al., 2014). To meet this predicted demand, crop varieties with higher yield, better adaptability to the changing climate as well as more tolerant to biotic and abiotic stresses will need to be developed in the next decade on an urgent basis.

Unfortunately, crop research and development, particularly crop genetics which has been focused on only a few crops such as wheat and maize which are historically grown in the temperate regions of the world, mainly Europe, North America and Central Asia. Despite enhancement of crop yield potential via conventional breeding methods like hybridization and mutation breeding, the actual crop yield seems to be approaching a plateau in recent decades (Mann, 1999; Ansari et al., 2017). Moreover, yield stagnation has been reported in some of the world's most intensive cropping systems such as rice in East Asia, maize in South Europe and wheat in Northwest Europe. One way of overcoming the limitations of the conventional breeding technologies is the use of biotechnology in crop improvement.

In recent years, sequence-specific genome editing technologies were found to be useful tools for crop improvement (Georges and Ray, 2017). In particular, the clustered regularly interspaced short palindromic repeats (CRISPR)/CRISPR-associated protein9 (Cas9) genome editing technology (CRISPR/Cas9)^1^ has so far shown the greatest promise for addressing emerging challenges in agriculture. The details of CRISPR/Cas9 technology are described and illustrated in some recent reviews (Sander and Joung, 2014; Westra et al., 2014; Bortesi and Fischer, 2015; Ma et al., 2016; Musunuru, 2017). This technology can be used to modify almost any genomic sequence to achieve desired traits in organisms including plants, with the only known limitation being the availability of the protospacer adjacent motif (PAM) sequence. Compared to other genome editing tools such as zinc finger nucleases (ZFNs) and transcriptional activator-like effector nucleases (TALENs), CRISPR/Cas9 is easier, more cost-effective, precise and is highly efficient even at multiplex genome editing (Wang M. et al., 2018). Applications of CRISPR/Cas9 in genome editing of plants is one of the most rapidly emerging technologies in biosciences. More importantly, the CRISPR/Cas9 is becoming a user-friendly tool for development of non-transgenic genome edited crop plants to cope up with changing climate and ensure future food security. This review updates our current knowledge and potentials of CRISPR/Cas9 for improvement of crops cultivated in tropical climates to gain resiliency against emerging pests and abiotic stresses. The purpose of this review is to provide updated information on the use of CRISPR/Cas9 in improvement of tropical crops as well as crops that are cultivated in tropical climate such as rice and wheat. In addition, we also focus on how CRISPR/Cas9 can be applied to improve crops that are better adapted to changing climate, emerging diseases, and improve product quality and address challenges pertaining to tropical crops.

## Progress of CRISPR/Cas9 technology in improvement of tropical crops

Emerging evidences are showing rapid expansion of CRISPR/Cas9 in a variety of field including agriculture. This section updates and highlights the application of CRISPR/Cas9 technology in improvement of tropical crops. Genome editing at target sites by specific nucleases has shown great promise for improvement of crop plants to meet the growing global demands of food. This technology offers an opportunity for biotechnologists to develop a sustainable and prolific agricultural system in terms of improving yield, abiotic stress tolerance, enhancing resistance to diseases and pests, and modification of plants for product quality. In the past, crop plants have been modified by conventional and modified plant breeding methods. These techniques are falling out of favor due to the requirement of longer time, problems of compatibility among the species, and the ever diminishing genetic variation of plants (Chen and Gao, 2014). In this regard, crop improvement requires genome editing with CRISPR/Cas9 technology to modify plant traits or add novel characteristics within a short period of time (Zhang and Zhou, 2014). Currently, CRISPR/Cas9 is also being used to produce site specific mutagenesis or targeted transcriptional regulation in several tropical crops. Meanwhile, CRISPR/Cas9 technology have been used in many crop plants cultivated in the tropical regions. The trends of application of the CRISPR/Cas9 technology for improvement of crop plants in tropical climates within the last 5 years are phenomenal, and it indicates a rapid large-scale application of it in addressing emerging challenges in production of crops in tropical climates.

## Improving yields and quality of crops cultivated in tropical regions using CRISPR technology

Crop yields are becoming increasingly vulnerable due to adverse climatic changes and deterioration of air quality and health of soils. To improve crop yield in the changing climate, scientists are looking for ways to engineer plants that can survive harsh and unpredictable environments and yield more. To achieve these targets, investigating the potential CRISPR/Cas9 to boost crop yields is becoming intense. By using CRISPR/Cas9 technology, a group of scientists at Cold Spring Harbor Laboratory (CSHL) precisely engineered the promoter sequence of quantitative genes in tomato (Rodríguez-Leal et al., 2017). By making small changes in the promoter regions in genes that control quantitative traits such as *LOCULE NUMBER* (control fruit shape and size), *FASCINATED* (responsible for large fruit size), *COMPOUND INFLORESCENCE* (control flower proliferation) and *SELF PRUNING* (control flowering time and hence growth habit) in tomato, researchers generated a wide range of new alleles that improved fruit shape, size as well as plant architecture (Rodríguez-Leal et al., 2017). This study demonstrates that CRISPR/Cas9 can be used to generate novel variations in plants where naturally existing variations are not available or has been greatly diminished. In rice, Xu et al. (2016) simultaneously mutated three genes (*GW2, GW5, TGW6*) that negatively regulate seed size. By mutating these genes in rice, they were able to increase seed size significantly (up to 30% in triple mutants). The CRISPR/Cas9-mediated mutation of *CLVTA3* genes in *Brassica napus* has resulted in higher seed number per silique as well as increased grain weight. Wang W. et al. (2018) recently applied CRISPR/Cas9 to increase the seed size in wheat. They knocked out the function of all homeologs of *TaGW2*, a gene which is known as negative regulator of seed size. Similarly, transgene-free low-gluten wheat has recently been engineered with CRISPR/Cas9 by Sánchez-León et al. (2018). Previous studies have shown that functionally active *TaGW2* gene of A genome negatively regulate grain weight and width (Su et al., 2011; Yang et al., 2012; Hong et al., 2014; Jaiswal et al., 2015; Simmonds et al., 2016) as well as grain length (Yang et al., 2012; Simmonds et al., 2016). When functionally active copies of *TaGW2* were mutated from the A, B and D genomes, the total grain weight (27.7%), grain width (10.9%), and grain length (6.1%) were significantly increased (Wang W. et al., 2018). This result shows that CRISPR/Cas9 can successfully be applied to engineer plants for higher yields. It would be interesting to see whether mutation of orthologous copies of *TaGW2* in rice or maize changes in the size and weight of seeds. These success stories indicate that CRISPR/Cas9 has high promise for the improvement of various traits of crop plants. It is expected that application of CRISPR/Cas9 in crops cultivated in tropical regions will significantly improve yield in near future and will be the key tool for plant biotechnologists to engineer crop plants for ensuring greatly demanding food security.

In addition to increase yield, CRISPR/Cas9 has also been used to improve quality of crops cultivated in tropical climates. Carotenoids play an important role in many physiological processes in plants and the phytoene desaturase genes encode important enzymes in the carotenoid biosynthesis pathway. Phytoene desaturase genes, *RAS-PDS1* and *RAS-PDS2* were recently mutated by the application of CRISPR/Cas9 with a 59% success rate in bananas (Kaur et al., 2018). This finding indicates that CRISPR/Cas9 can be used for the modification of quality of bananas, and a further improvement may just be on the horizon. Cassava is one of the most studied tropical crops and is an essential source of energy in tropical regions. Using CRISPR/Cas9 technology, *MePDS* mutants were generated in cassava, which exhibited albino or partial albino at cotyledonary-stage somatic embryos (Odipio et al., 2017). This phenotype was observed in over 95% of the mutant cassava. More importantly, the somatic embryo lines successfully produced plantlets with the mutations (22–47% success rate; Odipio et al., 2017). These findings suggest that CRISPR/Cas9 could be applied for the targeted improvement of this important tropical food crop. On the other hand, several groups were successful in generating mutations or deletions in one of the tropical cash crops, cotton with the target of *MYB25, GFP, GhVP, GhCLA1*, or *GhARG* genes (Chen X. et al., 2017; Janga et al., 2017; Li C. et al., 2017; Wang P. et al., 2017; Wang Y. et al., 2017). Gao and co-workers also demonstrated that *GhPDS* and *GhEF1* are targeted easily by CRISPR-mediated multiple mutations (Gao et al., 2017), supporting the idea that modification of several functions of cotton (or other tropical crops) at a time is possible, and thus attain a greater variety of options for the quality management and resistance to external stresses. Therefore, CRISPR/Cas9 could be used as an effective tool for the improvement of tropical crops and more success stories are expected to come soon.

## Development of abiotic stress tolerant crop plants in the tropics by CRISPR/Cas9

Abiotic stresses such as soil salinity, drought, heat stress etc. are now major threats to crop production that significantly limit yield of crops worldwide. The effect of these stresses is more severe in the tropical region than the temperate. One of the major abiotic stresses in tropical countries is high temperature. Global warming worsens the effect triggering several other types of abiotic stresses including drought. Drought is also another major abiotic stresses limiting crop production worldwide. The phytohormone ethylene is well-known to govern plant response to various abiotic stresses including drought (Kawakami et al., 2010) and heat (Hays et al., 2007).

Recently, it has been reported that 21 *KUP* genes in cassava are upregulated after exposure to several abiotic stresses including salt, osmosis, cold, drought, and H_2_O_2_. It was also found that differences in cassava genotypes influenced drought resistance through differential expression of *KUP* genes (Ou et al., 2018). A genome-wide study has revealed that *MAPKKK* genes play important roles in the tissue development of cassava and its resistance to drought stress (Ye et al., 2017). African oil palm is an important source of oil in tropical countries, which is highly sensitive to cold weather but has good resistance to salt and drought. It was found that *EgWRKY* genes of oil palm had showed tissue-specific expression patterns with higher levels under cold stress. It is now well recognized that almost all the *EgWRKY* genes are upregulated under abiotic stresses, and the authors suggest that the expression of *EgWRKY* genes in African oil palm play important roles in the responses to abiotic stresses (Xiao et al., 2017). Although CRISPR/Cas9 has not yet been applied for the improvement of most of the tropical crops, opportunity does exist to apply the technology for genetic modification of numerous tropical crops to increase yield and improve product quality, abiotic stress tolerance, and enhancing the resistance to destructive diseases and pests.

## CRISPR/Cas9 genome editing for addressing emerging diseases and pests in crops cultivated in tropical regions

Crop diseases caused by fungi, bacteria, oomycetes, viruses, and other microorganisms have increased in frequency in recent years and are enduring threat to global food security (Fisher et al., 2012). Emerging plant diseases are increasingly recognized as an important component of worldwide threat to food security. Despite modern agricultural practices, diseases of major food crops are responsible up to 15% pre-harvest yield losses. Various insect pests also cause significant crop losses worldwide. The CRISPR/Cas9-mediated genome editing technology has opened a new opportunity for rapid development of disease resistant crop varieties by either stacking of disease resistant (*R*) gene(s) or disruption/deletion of susceptibility (*S*) genes. Some success stories of CRISPR/Cas9-mediated genome editing for fungal, bacterial and viral disease resistant crop plants are reviewed in this section with a brief discussion on further potential use of this technology (Table 1).

**Table 1:** Some examples of CRISPR/Cas9-mediated genome editing in crop plants cultivated in the tropical climates for development of tolerance to abiotic and biotic stresses.

**Crop**	**Target gene(s)**	**Target traits**	**Type of edit**	**Results**	**References**
Banana	Phytoene desaturase	Trial for CRIPSR	Gene disruption	Decreased chlorophyll and total carotenoid contents	Kaur et al., 2017, 2018
Cassava	Phytoene desaturase	Trial for CRIPSR	Gene disruption	Observation of albino phenotype	Odipio et al., 2017
Cassava	*elF4E* isoforms *nCBP-1* & *nCBP-2*	Resistance to cassava brown streak disease	Gene disruption	Elevated resistance to cassava brown streak disease	Gomez et al., 2017
*Theobroma cacao*	*TcNPR3*, a suppressor of the defense response	Resistance to the cacao pathogen *Phytophthora tropicalis*	Gene disruption	Increased resistance to infection with the cacao pathogen *Phytophthora tropicalis*	Fister et al., 2018
Cotton (*Gossypium hirsutum*)	CLCuD IR and Rep regions	Resistance to cotton leaf curl disease	Viral gene disruption	Targeted cleavage of mixed infections by multiple viruses and associated DNA satellites, such as CLCuD-complex	Iqbal et al., 2016
Rice	*OsSWEET11*, *OsSWEET14* (rice bacterial blight susceptibility genes)	Resistance to bacterial blight	Promoter disruption	The promoter of the blight susceptibility gene was disrupted	Jiang et al., 2013
Rice	*OsERF922* (ethylene responsive factor transcription factor)	Resistance to rice blast	Gene disruption	Resistance to *M. oryzae* was enhanced	Wang et al., 2016
Wheat	*TaMLO-A1, TaMLO-B1* and *TaMLO-D1*	Resistance to powdery mildew	Gene disruption	The number of mildew microcolonies formed on the leaves was significantly reduced against the control and no apparent fungal growth was observed on the leaves of edited plants	Wang et al., 2014
Wheat	*TaDREB2* and *TaERF3*	Trial for CRISPR	Gene disruption	Provide a deep insight about their functioning in abiotic stress response	Kim et al., 2018

Rice is one of the most cultivated cereal crops in the tropical climate and it suffers from various diseases such as blast which can cause significant yield losses worldwide. Blast disease, caused by the filamentous fungus *Magnaporthe oryzae*, is considered as one of the deadliest rice diseases that has recently expanded its host range to other cereal crops such as wheat, barley, oat and millet (Sakulkoo et al., 2018). This disease is destroying food supplies to the extent that the lost production could feed hundreds of millions of people. Increased global trade, climate change, and the propensity of this pathogen to occasionally jump from one grass host to another, have resulted in increased incidence of blast diseases (Inoue et al., 2017). Wang and colleagues have developed mutagenized rice lines possessing enhanced blast resistance using CRISPR/Cas9 technology (Wang et al., 2016). They engineered the CRISPR/Cas9 vector (pC-ERF922) to target the rice *OsERF922* gene, which negatively regulates blast resistance of rice (Liu et al., 2012). In this process, pC-ERF922 was delivered into calli derived from seeds of blast susceptible rice variety Kuiku131 by *Agrobacterium*-mediated transformation with a mutagenic frequency of 42.0% in T_0_ transgenic plants. Edited plants that harbored the desired modification in the *OsERF922* were identified in the T_1_ and T_2_ segregating populations. The edited lines showed significantly enhanced blast resistance compared with wild-type. This study provided a successful example of improving PTI to enhance rice blast resistance using CRISPR/Cas9. Similarly, disruption of rice bacterial blight susceptibility genes, *OsSWEET11* and *OsSWEET14* using CRISPR/Cas9 has been achieved (Jiang et al., 2013). Recently, Ma et al. (2017) used CRISPR/Cas9 to knockout the function of *OsSEC3A* gene in rice and demonstrated that rice plant that lacks a functional copy of *OsSEC3A* provide better defense response as well as enhanced resistance to the fungal pathogen *Magnaporthe oryzae*, which causes rice blast disease.

Wheat is another important crop which is also widely cultivated in tropical areas. Wheat is a critical staple food providing 20% of the calories and over 25% of the protein consumed by humans (FAOSTAT, 2017; http://faostat.fao.org). Like rice, the wheat crop is susceptible to various diseases including blast. Wheat blast is a fearsome fungal disease caused by the *Triticum* pathotype of *M. oryzae* (MoT), which has been posing serious threat to wheat production since its first emergence in Paraná State of Brazil in 1985 (Igarashi et al., 1986; Kohli et al., 2011). In February 2016, an outbreak of wheat blast occurred in eight districts of Bangladesh, for the first time outside of South America (Callaway, 2016; Islam et al., 2016). It devastated more than 15,000 hectares of wheat with yield loss up to 100%. Within a year, it spread to new areas in Bangladesh and also in West Bengal of India (Bhattacharya and Pal, 2017). The Mexico-headquartered International Maize and Wheat Improvement Centre (CIMMYT) has reported that the consequences of a wider outbreak of the disease in South Asia could be devastating to a region of 300 million undernourished people whose inhabitants consume over 100 million tons of wheat each year (Collis et al., 2016). Considering the existing favorable environments for MoT in many countries in Asia, Africa and some states in the USA and also due to climate change, we fear that this worrisome enemy of major food crops may introduce and spread to the new wheat growing areas in future.

The ability of this blast fungus to evolve into new races is responsible for the limited success in controlling through breeding for resistance (Valent et al., 1991; Strange and Scott, 2005). Resistant resources against MoT are limited. The spread of the pathogen to some of the world leading wheat growing Asian countries such as India, Pakistan, and China could be catastrophic (Index Islam et al., 2016; Mundi, 2016). Mutation of genes associated with blast disease susceptibility (*S*-genes) may lead to enhanced wheat blast resistance. This strategy is emerging as an alternative to the “standard” plant breeding approach of introgressing major disease resistance (*R*-genes) (van Schie and Takken, 2014). It seems a practical proposition because of the development of genome editing methods that can be applied to cereal crops and which can simultaneously mutate all copies of the gene in polyploid genomes (Shan et al., 2014; Wang et al., 2014; Zhang et al., 2016; Sánchez-León et al., 2018). Over the years of research, it has been apparent that there are genes associated with enhanced susceptibility to rice blast disease. These represent a variety of gene functions which appear to be negative regulators of plant immunity responses, or which encode host proteins that are required by pathogens to help facilitate their entry and spread within plant tissue. A classic example of a susceptibility factor acting in this way is the barley *Mlo* locus, which is widely deployed as a recessive disease resistance gene (Büschges et al., 1997). When these genes are mutated, they lead to increase resistance to the disease. More than twenty rice *S*-genes (e.g., *OsRAC4/5/B, OsWAK112d, OsMAPK5, OsWRKY28/76, OsERF922, OsGF14b, SPL11, OB-fold gene, OsPLDbeta1*, and *OsSSI2*) have been characterized (Xiong and Yang, 2003; Zeng et al., 2004; Jung et al., 2006; Vega-Sánchez et al., 2008; Yara et al., 2008; Jiang et al., 2009; Yamaguchi et al., 2009; Chen et al., 2010; Delteil et al., 2012, 2016; Grand et al., 2012; Liu et al., 2012, 2016; Chujo et al., 2013; Yokotani et al., 2013; Xie et al., 2014; Wang et al., 2016). An example of a blast *S*-gene function is provided by some members of the WRKY transcription factor family. In rice, WRKY proteins comprise a complex transcriptional regulatory cascade that is important for the host response to rice blast disease. This network of transcription factors can act either positively or negatively to modulate the host immunity response, including response to pathogen-associated molecular patterns (PAMP) elicitors, jasmonic acid (JA) signaling or other cellular functions that impact the outcome of the interaction. Among the WRKY proteins, *OsWRKY28*, is a PAMP-responsive repressor that negatively regulates innate immune responses in rice against the rice blast fungus (Chujo et al., 2013). Another example is *OsMAPK5*, a MAP kinase that mediates immune signaling and represses defense gene expression, thereby negatively regulating rice blast resistance. Remarkably, loss of function of mutations in *OsMAPK5* lead to enhanced resistance to the blast disease in rice (Xiong and Yang, 2003; Xie et al., 2014). Wang et al. (2014) developed powdery mildew resistant wheat by disruption of *TaMLO-A1, TaMLO-B1*, and *TaMLO-D1* genes in the wheat genome by the CRISPR/Cas9. Therefore, it may be possible to achieve resistance to wheat blast by mutation in wheat orthologs of several rice susceptibilities (*S*-) genes as means of mitigating emerging wheat blast problems in a short period of time. We foresee CRISPR/Cas9 edited non-transgenic homozygous *S*-genes mutants for durable blast resistant wheat varieties.

## CRISPR/Cas9 genome editing for addressing some diseases and pests of tropical crops

Cassava is a staple food of African small-hold farmers. Several diseases such as brown streak, mosaic and bacterial blight cause significant yield losses in cassava. CRISPR/Cas9 can be used to develop resistant varieties against these diseases resulting in improving crop yield and livelihood of resource poor African farmers (Bart and Taylor, 2017). Editing of *Phyteone desaturase gene* in cassava using CRISPR/Cas9 showed that it could be an efficient genome editing tool for cassava (Odipio et al., 2017). Cassava brown streak disease is a major constraint on cassava yield in East and Central Africa. It is caused by the family Potyviridae which require the interaction of viral genome-linked protein (VPg) and host eukaryotic translation initiation factor *4E(elF4E)* isoforms. Simultaneous CRISPR/Cas9-mediated editing of cassava elF4E isoforms nCBP-1 and nCBP-2 has shown elevated resistance to cassava brown streak disease (Gomez et al., 2017).

Papaya is a highly nutritious and medicinally important tropical fruit crop which is susceptible to viral pathogens. Transgenic papaya varieties such as “SunUp” and “Rainbow” developed against a major viral pathogen, *Papaya ringspot virus* (PRSV), has significantly promoted the papaya industry in Hawaii. However, more than two decades of Hawaiian experience of transgenic papaya cultivation did not replicate in other countries for various biotic and abiotic factors. Major factors include rapid emergence of recombinant strains, virus-encoded PTGS suppressors, sequence homology between transgene and infecting *PRSV CP* gene, transgene copy number, and emergence of new viruses (Hamim et al., 2018). These problems can largely be solved by the stacking of multiple virus resistant genes in papaya through CRISPR/Cas9. Alternatively, CRISPR/Cas9-mediated mutations of *S*-genes in the papaya plant could also confer resistance to PRSV (Green and Hu, 2017). Papaya plants contain high level of papain, a cysteine protease which mediate plant defense against pathogen and insect attack. However, *Phytopthora palmivora*, a fungal pathogen can infect all parts of papaya plant in spite of having high amount of papain in the infected parts. This suggests that *P. palmivora* contains cysteine inhibitors, which enable them overcoming papain mediated resistance. By using CRISPR/Cas9, researchers at the University of Hawaii Manoa have identified a cysteine protease inhibitor, cystatin (PpalEPIC8) which plays major role in suppressing the defense mediated by papain. Mutation of *PpalEPIC8* in *P. palmivora* has resulted in increased sensitivity to papain suggesting that it plays a major role in *P. palmivora* virulence by inhibiting papain (Gumtow et al., 2018). These findings suggest that CRISPR/Cas9 technology in combination with *Agrobacterum*-mediated transformation could easily be applied for the development of homozygous mutants in most destructive pathogens, oomycetes, which is either impossible or difficult by conventional methods. The success in application of CRISPR/Cas9 in easy generation of mutants of *P. palmovira* opens a new window for understanding the molecular basis of plant- oomycete interactions that ultimately help to develop resistant crop plants against this (oomycete) extremely destructive class of phytopathogens. *Theobroma cacao (T. cacao)*, the tropical tree which produces cocoa beans, is the centerpiece of the multi-billion-dollar chocolate industry and is a vital export for many developing countries. Fister and colleagues have targeted a universal defense suppressor gene *TcNPR3NPR3* in *T. cacao* and obtained mutagenized tissues after transient incorporation of CRISPR/Cas9 components. The edited tissues demonstrated increased resistance as demonstrated by less infection and up-regulated expression of PR genes (Fister et al., 2018). A large-scale application of CRISPR/Cas9 technology based on these protocols might help to develop resistant crop plants against the major crop pests in the tropical regions.

## Challenges pertaining to application of CRISPR/Cas9 in tropical crop plant

Although genomes of many tropical crop plants have been sequenced, the function of the vast majority of genes remains unknown. In other words, we have not reached the level of complete understanding of the functions of the major genes in tropical plants. However, recent genome sequencing technology and genome-wide association studies (GWAS) allow us to predict the function of many genes, which confer resistance to diseases in tropical fruit and other crops. Using next generation sequencing (NGS) and GWAS datasets, Kayondo et al. (2018) has confirmed that the resistance of cassava to CBSD (cassava brown streak disease) is polygenic and environment-dependent. More importantly, they have identified the candidate resistance gene *NBS-LRR* on chromosome 11 (Kayondo et al., 2018). A systematic analysis has also identified 4 *MeDELLAs* as cassava bacterial blight resistant genes (Li et al., 2018). Moreover, 77 bZIP transcription factor has been identified in cassava using RNA-Seq (Hu et al., 2016), while *MebZIP3* and *MebZIP5* have been identified as resistance enhancing genes to cassava bacterial blight (Li X. et al., 2017). However, data regarding correlation between tropical disease and gene mutation or modification of gene expressions is still limited. To make CRISPR/Cas9 more useful in tropical plant breeding, further studies on plant gene function and their regulatory elements are needed. New discoveries unraveling the role of tropical plant genes and improvement of CRISPR/Cas9 in the near future should facilitate overcoming various plant breeding problems in tropical areas. A list of potential genes that can be targeted by the CRISPR/Cas system is given in the Table 2.

**Table 2:** Tropical plant genes that can be edited by the CRISPR/Cas9 technology to improve plant tolerance to the abiotic and biotic stresses.

**Crop**	**Target gene(s)**	**Target traits/stress**	**References**
Avocado	*PAL* and *LOX*	Anthracnose disease resistance	Bill et al., 2017
Avocado	*PaNPR2* and *PaNPR4*	*Phytophthora cinnamomi* resistance	Backer et al., 2015
Banana	*MaSWEET-1a, MaSWEET-4b, MaSWEET-14b, MaSWEET-4c, MaSWEET-14c, MaSWEET-4d, MaSWEET-14d*, and *MaSWEET-14h*	Foc 4 TR4 and abiotic stresses (cold and salt) resistance	Miao et al., 2017a
Banana	*MaATG8s*	*Fusarium oxysporum* f. sp. *cubense* (Foc) resistance	Wei et al., 2017b
Banana	*Hrap, Pflp*	*Xanthomonas campestris* pv. *musacearum* resistance	Tripathi et al., 2010, 2014; Namukwaya et al., 2012
Banana	*MaAPS1* and *MaAPL3*	Abiotic stresses (cold and salt) and *Fusarium Oxysporum* f.sp. *cubense* (Foc) Tropical Race 4 (TR4) resistance	Miao et al., 2017b
Cassava	*RXam1*	*Xanthomonas axonopodis* pv. *manihotis (Xam)* strain-specific resistance to *XamCIO136*	Díaz Tatis et al., 2018
Cassava	*MeWRKY20-MeATG8a/8f/8h* (*MeATG8a, MeATG8f, MeATG8h*)	Cassava bacterial blight (CBB), caused by *Xanthomonas axonopodis* pv. *manihotis (Xam)* resistance	Yan et al., 2017
Cassava	*MeDELLAs*	Cassava bacterial blight (CBB) resistance	Li et al., 2018
Cassava	*MebZIPs (MebZIP3* and *MebZIP5*)	Cassava bacterial blight (CBB) resistance	Li X. et al., 2017
Cassava	*MeRAV1* and *MeRAV2*	Cassava bacterial blight (CBB) resistance	Wei et al., 2017a
Cassava	*MeKUPs*	Abiotic stresses (salt, osmosis, cold, drought) resistance	Ou et al., 2018
Cassava	*MeMAPKKK*	Abiotic stress (drought) resistance	Ye et al., 2017
Coconut	*PTI5*	Root wilt disease (RWD) resistance	Verma et al., 2017
Coconut	*NBS-LRR type RGAs*	Coconut root (wilt) disease resistance	Rajesh et al., 2015
Cotton	*GhPIN1–3* and *GhPIN2*	Abiotic stress (drought) resistance	He et al., 2017
Cotton	*GhRDL1*	Abiotic stress (drought) resistance	Dass et al., 2017
Cotton	*GhERF-IIb3*	Bacterial blight caused by *Xanthomonas citri* pv. *malvacearum (Xcm*) resistance	Cacas et al., 2017
Date palm	*Pdpcs* and *Pdmt*	Abiotic stress (Cd and Cr) resistance	Chaâbene et al., 2018
Date palm	*Pdpcs* and *Pdmt*	Abiotic stress (metals) resistance	Chaâbene et al., 2017
Papaya	*CpDreb2*	Abiotic stresses (drought, heat and cold) resistance	Arroyo-Herrera et al., 2016
Papaya	*CpRap2.4a* and *CpRap2.4b*	Abiotic stresses (heat and cold) resistance	Figueroa-Yañez et al., 2016
Sugarcane	*ScGluA1*	Smut (*Sporisorium scitamineum*) resistance	Su et al., 2013
Sugarcane	*ScCAT1*	Smut (*Sporisorium scitamineum*) resistance	Su et al., 2014
Sugarcane	*ScAPX6*	Abscisic acid (ABA), methyl jasmonate (MeJA), and copper (Cu) stress resistance	Liu et al., 2017
Sugarcane	*ScGluD2*	Smut and abiotic stresses (salt and heavy metal) resistance	Su et al., 2016
Sugarcane	*ScChiIV1* and *ScChiVI1*	Smut (*Sporisorium scitamineum*) resistance	Su et al., 2015
Sugarcane	*ScNsLTP*	Abiotic stresses (drought and chilling) resistance	Chen Y. et al., 2017

As shown in Table 2, expression of *MaAGPase* and *MaSWEETs* gene*s* are increased following exposure to abiotic stressors including salt, cold, and drought in bananas. A similar effect was noted when banana plants are infected with *Fusarium oxysporum f.sp. Cubense* (Foc) Tropical Race 4 (TR4) (Miao et al., 2017a,b). In grapes and citrus, *P. viticola* and Huanglongbing (HLB) infection both directly increase AGPase activity leading to enhanced starch biosynthesis (Kim et al., 2009; Gamm et al., 2011). Since transcriptional up-regulation of *MaAGPase* genes occurs in response to Foc TR4 infection, these genes may play a role in modulating the response to fungal infections in banana (Miao et al., 2017b). As such, CRISPR/Cas9-mediated knockout of these genes may lead to Foc TR4 infection resistance. Interestingly, *MaSWEETs* expression increases with exposure to cold, salt and osmotic stress. Most *MaSWEETs* genes are upregulated when plants are exposed to these stressors (Miao et al., 2017a). Accordingly, *MaSWEETs* genes play an important role in bananas' response to these stressors (Miao et al., 2017a). Bioengineered banana expressing sweet pepper *Hrap* and *Pflp* genes have demonstrated complete resistance to *Xanthomonas campestris* pv. *musacearum* (Tripathi et al., 2010, 2014; Namukwaya et al., 2012). Host-induced gene silencing (HIGS) has also been reported in bioengineered banana. HIGS of *Fusarium oxysporum* genes moderate Fusarium wilt in transgenic banana (Ghag et al., 2014). CRISPR/Cas9 could therefore modify banana gene expression to improve abiotic stress tolerance as well as enhance resistance to various diseases and pests. Ardi et al. (1998) showed that epicatechin is involved in the resistance of avocados to anthracnose disease. The up-regulation of *phenylalanine ammonia-lyase* gene expression and down-regulation of *lipoxygenase* gene expression enhances epicatechin biosynthesis (Bill et al., 2017). In fact, epicatechin directly governs the concentration of antifungal diene compound (AFD) by regulating lipoxygenase activity (Prusky et al., 1992). AFD is the most active antifungal compound found in avocado. It alters the dormancy of *Colletotrichum gloeosporioides* in unripe fruit. In the near future, CRISPR/Cas9 technology may modify genes involved in AFD biosynthesis to increase resistance of avocado to fungal infection. Additionally, Verma's group successfully generated transcriptomic databases of genes associated with root wilt disease (RWD) including the NBS-LRR domain, PR1, PR4, pathogenesis-related genes transcriptional activator PTI5-like gene, thaumatin-like protein, HSP70 and glutathione *S*-transferase. Accordingly, modifying these coconut genes with CRISPR/Cas9 may lead to improved quality management as well as increase resistance to external stressors.

A prerequisite for successful CRISPR/Cas9-mediated plant gene editing is the availability of efficient delivery methods for the gene editing components and a satisfactory plant regeneration system (Altpeter et al., 2016; Ran et al., 2017). Although many successful reports of CRISPR-mediated plant gene editing have now been published, there are a number of factors which affect efficiency. The frequency of gene editing in tropical plant species will depend on whether or not these conditions such as efficient gRNA designing, assembling multiple gRNA cassettes, efficient delivery of Cas9 and gRNA vectors or ribonucleoproteins (RNP), selection and regeneration of edited plantlets, and efficient detection of the gene editing event are optimal. All delivery systems have advantages and disadvantages. The PEG-mediated delivery of CRISPR reagents using transient protoplasts system is very useful for gRNA validation but only a very small number of plant species can be regenerated from protoplasts (Woo et al., 2015). On the other hand, haploid microspores, or immature pollen grains can be easily isolated from many different species in a relatively short period of time, and used as targets for transformation and early screening of gene editing events. Although a functional microspore-based gene editing system using electroporation, biolistic and *Agrobacterium*-mediated delivery has several advantages compared to conventional plant transformation-based systems, it has very low efficiency regarding regeneration of microspore edited plants (Brew-Appiah et al., 2013; Bhowmik et al., 2018).

The *Agrobacterium* and biolistic mediated delivery of the CRISPR reagents also have several drawbacks. It requires time, and labor for preparation of a large number of explants and regeneration of CRISPR edited plants through tissue culture may also take a long period (Liang et al., 2018). In addition, *Agrobacterium*-mediated transformation is typically genotype specific, and the efficiency of the biolistic gene transfer needs optimization of several parameters such as particle type, size, quantity and acceleration, DNA amount and structure during particle coating, tissue type, and pre-treatment (Altpeter et al., 2016). Therefore, the development of efficient and reproducible delivery systems for genome editing would be an important step in moving the powerful CRISPR/Cas9 for routine use in tropical crop species.

In order to improve plant transformation through CRISPR/Cas9, several approaches such as optimization of the promoters to drive and express Cas9 and utilization of different fluorescent reporters and selection markers (Wang et al., 2015; Yan et al., 2015; Kaur et al., 2018) have recently been evaluated. To test whether the CRISPR/Cas9 can be used to precisely edit an endogenous gene in banana, Kaur et al. (2018) designed a specific gRNA from the most conserved region (5th exon) of the *RAS-PDS* genes and inserted it into the binary vector pRGEB31 under rice *snoRNA U3* promoter using a *Bsa*I restriction site. This vector also contained a *Cas9* endonuclease-encoding sequence driven by the dual *CaMV 35S* promoter and hygromycin *phosphotransferase* (*HPT*II) selection marker genes. Authors have reported 59% mutation efficiency in *RAS-PDS* in the banana cv. Rasthali genome using hygromycin for the selection of positive transformed plants. Another approach by overexpressing candidate genes such as *Baby boom* (*Bbm*) and *Wuschel2* (*Wus2*) from maize (*Zea mays*) has increased transformation frequencies in maize, sorghum (*Sorghum bicolor*), sugarcane (*Saccharum officinarum*), and indica rice (*Oryza sativa* ssp. *indica*) (Lowe et al., 2016). Because of the advantage of being independent of genotype, this approach may be useful for advancing transformation efficiency and CRISPR editing in difficult to transfect tropical crop species. As CRISPR/Cas9-mediated plant genome editing still faces challenges, primarily not being able to establish a genotype-independent delivery method, extending and improving the transformation platform will help to deploy this technology for the improvement of tropical species.

## Conclusion

Many success stories on application of CRISPR/Cas9 in genome editing of tropical crops and crops cultivated in tropical regions have not been reported yet as compared to temperate crops. To have a greater impact on agriculture in tropical areas, further efforts are needed to optimize the CRISPR/Cas9 protocols for making it more user-friendly and freely accessible for research and practical applications. Development of an efficient transformation system for major tropical crops and crops such as Indica rice (Ishizaki, 2016) in tropical climates would facilitate the development of crops resilient to emerging pests and abiotic stresses. International collaboration through open data sharing and practice of open science are needed to rapidly tackle any emerging challenges in agriculture such as recent emergence of wheat blast disease in tropical areas of Asia. CRISPR/Cas9 mediated genome edited (deleted or disruption of undesirable genes/sequences) crop plants should be considered as non-GMO for rapid application and acceptance of this technology at the field level. We foresee the application of CRISPR/Cas9 technology in various crops revolutionize agriculture in a second green revolution to ensure food and nutritional security of the ever-increasing population of tropical countries.

## Author contributions

TI conceived, coordinated, wrote and edited the manuscript. EH, HT, and MH wrote, interpreted data and revised the manuscript. PB prepared surveyed literature, prepared graphs and wrote manuscript. RK, MS, KZ, and MR edited and revised the manuscript.

### Conflict of interest statement

The authors declare that the research was conducted in the absence of any commercial or financial relationships that could be construed as a potential conflict of interest.

## Footnotes

^1^CRISPR/Cas9 Technology In Rice And Wheat. Posted 8th March 2017 by Fabio Caligaris. http://www.global-engage.com/agricultural-biotechnology/crisprcas9-technology-rice-and-wheat/.
